# Contribution of BRCA1 and BRCA2 Germline Mutations to Early Algerian Breast Cancer

**DOI:** 10.1155/2016/7869095

**Published:** 2016-02-22

**Authors:** Sarra Henouda, Assia Bensalem, Rym Reggad, Nedda Serrar, Leila Rouabah, Pascal Pujol

**Affiliations:** ^1^Laboratory of Molecular and Cellular Biology, Faculty of Natural and Life Sciences, Mentouri Brothers University, Constantine, Algeria; ^2^Medical Oncology Department, EH Didouche Mourad, Constantine, Algeria; ^3^Faculty of Medicine, University of Constantine 3, Constantine, Algeria; ^4^Medical Oncology Department, Anti-Cancer Center of Setif, Setif, Algeria; ^5^Department of Medical Genetics, Montpellier Regional University Hospital Center, Montpellier, France

## Abstract

Breast cancer is the most common female malignancy and the leading cancer mortality cause among Algerian women. Germline mutations in the BRCA1 and BRCA2 genes in patients with early-onset breast cancer have not been clearly identified within the Algerian population. It is necessary to study the BRCA1/2 genes involvement in the Algerian breast cancer occurrence. We performed this study to define germline mutations in BRCA1/2 and their implication in breast cancer among young women from eastern Algeria diagnosed or treated with primary invasive breast cancer at the age of 40 or less who were referred to Anti-Cancer Center of Setif, Algeria. Case series were unselected for family history. Eight distinct pathogenic mutations were identified in eight unrelated families. Three deleterious mutations and one large genomic rearrangement involving deletion of exon 2 were found in BRCA1 gene. In addition, four mutations within the BRCA2 gene and one large genomic rearrangement were identified. Novel mutation was found among Algerian population. Moreover, five variants of uncertain clinical significance and favor polymorphisms were identified. Our data suggest that BRCA1/2 mutations are responsible for a significant proportion of breast cancer in Algerian young women.

## 1. Introduction

Breast cancer is the most common cancer among women both in more and less developed countries with faintly more cases in less developed countries [1]. It is the most recurrent cancer and the leading cause of cancer mortality in Algerian women [1, 2]. BRCA1 and BRCA2 are the major genes associated with hereditary breast cancer susceptibility [3, 4]. Women with deleterious mutations in these genes increase considerably an estimated cumulative lifetime risk for developing particularly breast and ovarian cancers. A significant proportion of this risk occurs in women under the age of 50 years [5]. According to literature [6], the average cumulative risks of developing breast cancer for BRCA1 and BRCA2 mutation carriers by the age of 70 years are 65% and 45%, respectively, and those of ovarian cancer are 39% and 11%, respectively. Lifestyle and environmental factors can modify the breast cancer risk range. Approximately 25% of breast cancer cases associated with a genetic predisposition are diagnosed before they reach the age of 30 [7]. Breast cancer elevated rates among women under 40 years in our country suggest a genetic factor contribution; we proposed that a considerable proportion of Algerian cases are attributed to these two susceptibility major genes. There is a little information on the frequency of BRCA1/2 mutations among Algerian population. In fact, the main objective of the present study was to investigate the contribution of BRCA1/2 mutation in early-onset breast cancer in Algerian women and to better understand the genetic risk factors associated with this disease.

## 2. Materiel and Methods

The population studied concerned forty Algerian primary invasive breast cancer cases selected from patients diagnosed at 40th or below referred to Anti-Cancer Center of Setif, eastern Algeria during the period 2013-2014, unselected for family history, to assess pathophysiological factors that may influence clinical outcome and prognosis including patient demographics, clinical presentation, treatments, and genetic spectrum. All patient detailed data regarding personal and family history of breast cancer were gathered on individual interview. Clinical and pathological characteristics were collected from medical records. Eligible patients had to be alive, be ≥18 years of age, and consent to genetic testing for BRCA1/2. All selected patients were informed about study objectives and they gave their signed informed consent for inclusion before they participated in the study. The study was conducted in accordance with the Declaration of Helsinki, and the protocol was approved by the Ethics Committee of Anti-Cancer Center of Setif (KsBR/S11/2012).

Forty blood samples were taken from 40 different families diagnosed with early-onset breast cancer stored in EDTA tubes; most samples were of 5 mL, to investigate the contribution of BRCA1/2 mutations in breast cancer among Algerian young women. Analysis consists of PCR-based, bidirectional Sanger sequencing of all translated exons and immediately adjacent intronic regions of the BRCA1/2 genes, as well as large rearrangement analysis by multiplex ligation-dependent probe amplification (MLPA) if a negative result was found of all BRCA1 (OMIM 113705/GenBank entry U14680) and BRCA2 (OMIM 600185/GenBank entry U43746) exons (Comprehensive BRACAnalysis® of Myriad Genetic Laboratory Munich, Germany). Technical aspects of this analysis have been previously described in detail [8–11]. The classification and interpretation of all variants identified in this assay reflect the current state of scientific understanding. A confirmation test has been performed for each individual. All mutations and variants are cited according to Human Genome Variation Sequence systematic nomenclature. Mutations were defined according to different categories: deleterious, suspected deleterious, variant of unknown significance, and variant-favor polymorphism [10]. In our study, we classified BRCA1/2 suspected deleterious mutations and deleterious mutations as a positive test result; we reported insignificant variant in a separate category. We classified a favor polymorphism or no mutation detected as negative test result. All mutations and genetic variants are named according to the convention of Beaudet and Tsui [12]. Nucleotide numbering starts at the first transcribed base of BRCA1 and BRCA2 based on GenBank entries U14680 and U43746, respectively. (Under these conventions, the two mutations commonly referred to as “185delAG” and “5382insC” are named 187delAG and 5385insC, resp.)

Statistical analyses in the present study were performed using SPSS version 20. This is the first published study analyzing complete genes BRCA1/2 using PCR, bidirectional Sanger sequencing, and MLPA techniques among young patients unselected for family history in this region.

## 3. Result

The mean age of all patients at diagnosis was 36.58 years (range 27–40, median: 38) and that for BRCA mutation carriers was 34.63 years (range: 28–40). Of the 40 patients, only one breast cancer case was detected by screening mammographic before breast cancer appearance. All patients were diagnosed with unilateral breast cancer. The mean clinical tumor size was 4.33 cm (1–12).

We analyzed BRCA1 and BRCA2 genes; a total of eight BRCA deleterious germline mutations were found in eight unrelated patients with frequency of 20% (8/40), four within BRCA1 and four within BRCA2; these mutations range from deletion or substitution of a single nucleotide to whole deletions of one or more exon of either BRCA1 or BRCA2. Mutations observed in our series included two large rearrangements.

The majority of BRCA mutation carriers included in this study were of urban and Kabyle origin (7/8), were married (7/8), were overweight or obese (6/8), had an elevated level (5/8), had no family history of breast cancer in 1st, 2nd, and 3rd degree (87.5%), had full-term pregnancies (87.5%), had two children, had oral contraceptive (87.5%), breastfeed their infants (62.5%), had a delay between the first signs of cancer and consultation varied from 2 to 6 months (75%), had their breast cancer diagnostic out of pregnancy or breastfeeding (87.5%), and had a nodule self-examination as revealing sign (75%). Moreover, BRCA-associated tumors are more likely to be uninodular (62.5%), mobile (38%), IDC (75%) followed by IDC associated with ILC (25%), occurring in the left breast compared with the right (62.5%), located in the upper outer quadrant (50%), and diagnosed in advanced stages (stage I: 12.5%; stage II: 25%; stage III: 50%). Most BRCA carriers in the present research were treated with mastectomy (87.5%) and had no hormonal therapy (62.5%). In addition, 50%, 50%, and 12.5% of BRCA mutation carriers were estrogen, progesterone, and human epidermal growth factor receptor-2 positive, respectively. 50% of tumors among carriers were of grade II, (37.5% and 12.5% were of grades III and I, resp.). Furthermore, 62.5% of tumors among carriers were associated with lymph node involvement (N+) and 25%: N−, the lymph node involvement was accompanied with capsular rupture in 37.5%. All BRCA carriers did not practice any physical activity, had no breast cancer screening, were found in families with site-specific breast cancer, had no metastasis at the moment of the diagnostic, and underwent adjuvant chemotherapy with treatments number varying from 6 to 8 (mean: 6.25).

The elevated mutation frequency in BRCA1/2 genes was observed among women diagnosed between 36 and 40 years (4/8), followed by those of 26 to 30 (3/8) and then those of 31 to 35. For BRCA carriers, the mean number of pregnancies was 3.43 (1–7), the mean duration of breastfeeding was 23 months (6–33), the mean delay between first signs of cancer and consultation was 6.33 months (2–15), and the clinical tumor size mean was 3.4 cm (1.5–6). The voluntary termination of a pregnancy had not been found in our series. Clinical characteristics of study population (age at diagnosis, at menarche, at first pregnancy, and at first use of oral contraceptive) are summarized in Table 1.

Overall, four distinct deleterious mutations within the BRCA1 gene were identified in patients tested.

The heterozygous germline BRCA1 mutation c.1817del is predicted to result in the substitution of proline for leucine at amino acid position 606 followed by a premature truncation of the BRCA1 protein at amino acid position 611. The heterozygous germline BRCA1 mutation c.4065_4068del is predicted to result in the substitution of asparagine for lysine at amino acid position 1355 followed by a premature truncation of the protein at amino acid position 1364. The BRCA1 mutation c.5332+1G>A is located 1 nucleotide downstream of exon 21; this mutation occurs within a consensus splice junction, and it is predicted to result in abnormal mRNA splicing. Finally, one large genomic rearrangement was identified in BRCA1 gene: del exon 2 results in a deletion of exon 2, which includes the BRCA1 translation start codon, it is also known as BRCA1 c.(-19−?)_(80+?)del.

Moreover, four different deleterious mutations were identified in BRCA2 gene: the BRCA2 mutation c.7654dupA is predicted to result in the substitution of isoleucine for asparagine at amino acid position 2552 followed by a premature truncation of the BRCA2 protein at amino acid position 2553. The BRCA2 mutation c.1528G>T is predicted to result in the premature truncation of the protein at amino acid position 510. The BRCA2 mutation del exons 19-20 results in the deletion of exons 19 and 20 of the BRCA2 gene, and finally the BRCA2 mutation c.6450del is predicted to result in the substitution of valine for phenylalanine at amino acid position 2151 followed by a premature truncation of the BRCA2 protein at amino acid position 2167. Genetic and histopathological characterizations of the Algerian carriers are summarized in Table 2.

All mutations found in our series were unique, detected only once. Additionally, four distinct variants of uncertain clinical significance were found within BRCA2 gene in four unrelated patients (c.7462A>G, c.1504A>C, c.5939C>T, and c.1627C>A). A favor polymorphism in BRCA1 was found in two unrelated patients: c.5117G>C (see Table 3).

## 4. Discussion

The contribution of BRCA1/2 mutations to both hereditary and sporadic breast cancer has not yet been thoroughly investigated in Algeria. There are few studies examining the Algerian BRCA status; thus knowledge about the prevalence of BRCA mutations in this population is insufficiently explored. A total of 40 breast cancer patients have been studied for germline mutation in both BRCA1 and BRCA2 genes to contribute to the clarifying of this situation; the entire BRCA1/2 were analyzed using a direct sequencing. Eight deleterious distinct mutations have been detected in eight unrelated patients providing an overall prevalence rate of 20% (four within BRCA1). According to [13], the frequency of BRCA1/2 mutations varies among populations; Tunisian and Moroccan populations have reported frequencies of 19.4% and 25.64% as reported by [14, 15], respectively. These differences are principally related to inclusion criteria of cases and their ethnicity. In this study, BRCA1 mutation rate is identical to BRCA2 mutation rate; this result may be due to the small number of patients analyzed but if this finding is confirmed in elevated sample, the BRCA1/2 mutations may contribute similarly to early-onset breast cancer in Algeria. To our knowledge, one novel deleterious mutation was detected within the BRCA2 gene, the heterozygous germline BRCA2 c.6450del mutation not previously reported in other populations; it was found in patient diagnosed at 30 years of age and without family history of breast cancer. The remaining variants identified in the present report were already described in the Breast Cancer Information Core Database (http://research.nhgri.nih.gov/bic/index.shtml). Mutations identified in our series were not found earlier in Algerian patients with sporadic or heredity breast cancer except the large rearrangement involving the BRCA2 gene caused the deletion of exon 2 reported by [2] and the heterozygous germline BRCA1 c.1817del mutation, suggesting may be an Algerian funder mutations. The variants of uncertain clinical significance identified here have been already found but not in Algerian population. The favor polymorphism c.5117G>C identified in BRCA1 gene had been detected in Algerian and Moroccan populations [16, 17] but it was reported as an unclassified variant. There was no strong evidence that this variant might be disease associated. It is likely that this variant may have a functional relationship with breast cancer development, but this remains to be more explored. Nonpathogenic and unclassified variants identified in our series were all identified among patients with negative BRCA result. The majority of BRCA1/2 mutations in this study consist of single base changes or deletions/insertions of small numbers of bases that result in protein truncation, disruption of messenger RNA processing, or amino acid substitutions that have significant impact on protein function. A minority of mutations in the* BRCA1*/*2* genes are large rearrangements consisting of deletions and duplications of one or more exons. This finding is in agreement with literature [18]. Our findings demonstrate that BRCA1/2 mutation carriers had no breast/ovarian strong family history, as has been reported by literature [19]. As shown in Table 2, in most BRCA carriers, there was no family history of breast cancer (2/8). Breast cancer family history was positive in two women found to have nonpathogenic mutations; one of them was diagnosed with uncertain clinical variant associated with favor polymorphism. Of the 32 patients with a negative test result, eight had a personal history of breast cancer before the age of 35 years. One patient had a familial history of breast and ovarian cancers, five women had a familial history for breast cancer only, and one participant had an ovarian cancer family history only (12.5% had a breast cancer family history in 1st, 2nd, and 3rd degree). Two interpretations may contribute to explaining this finding: the reported family histories of breast cancer in this study may not have been accurate and possibly there were some omissions on the one hand, and on the other may be the Algerian population has a particular composition given the low incidence of breast cancer compared with Western countries, revealing the greater contribution of genetic factors. We note that all pathogenic mutations found occur in patient with unilateral cancer, contrary to what was reported by [2, 20]. Our findings among Algerian younger patients in this region differ from those provided by the literature; these results should be confirmed on a higher sample.

For the forty breast cancers studied, the mean age at diagnosis was 34.62 years for BRCA1/2 positive and 37.06 years for BRCA negative cancers. Of the eight patients with a BRCA1/2 positive result, three had a personal history of breast cancer ≤ the age of 30 years. Of the 32 patients with BRCA1/2 negative result, four patients were found to have a variant of uncertain clinical significance; none of these patients had a personal history of breast cancer before the age of 35 years. These results demonstrate a younger age at diagnosis for BRCA positive compared to the all cases diagnosed in our study. Carrying a* BRCA* mutation increasing the age of diagnosis of breast cancer is well documented [2, 21]. All women selected for this study had a personal history of first breast cancer. The most common personal history among these women in general was personal breast cancer ≤ 38 years and the most common family history was none. In our current study, the median age at diagnosis for all breast tumors was younger than that for those in Europe and America [22]. Most of the BRCA mutations are estimated to occur in inactivated patients with urban and Kabyle origin. The high prevalence of BRCA1/2 reported here could be linked to Kabyle genetic elements of this population. The ethnicity of these patients may give them a different genetic composition. Urbanization and the sedentary lifestyle of the Algerian younger women (decline of age at first pregnancy and number of children, short duration of breastfeeding, lack of physical activity and obesity, etc.) play a role in early apparition of breast cancer and may modify the penetrance of BRCA mutation influencing the genetic risk to develop breast cancer because having the mutated gene does not mean developing breast cancer. Environmental and lifestyle factors can modify the risk of breast cancer in women with BRCA1/2 mutations. According to [6, 21, 22], it appears that the risk to develop breast cancer has increased in recent generation. Environmental and lifestyle factors can modify the risk of breast cancer in women with BRCA1/2 mutations. Age at breast cancer diagnosis seems to have an effect on the BRCA1/2 detection rate. The frequency of BRCA mutation carriers depends on the population studied and displays considerable variation that coincides with ethnic and geographical diversity [23]. To date the highest BRCA1 mutations frequency among breast cancer cases is in Bahamas [24]. Variation in rate of BRCA mutation can be explained by the difference in lifestyles and breast cancer risk factors. Of the nine triple-negative breast cancer diagnoses in the present study, four occurred in patient's carriers of BRCA1 mutations. This result is consistent with reports in the literature [25, 26]. In contrast, most BRCA2 carcinomas in the present study were associated with hormonal receptor and HER2 positive; our results are in good concordance with those obtained by [27]. 50% of BRCA mutations occurred in case diagnosed at 35 years of age or below and not with elevated number of relatives affected with breast or ovarian cancer contrary to other reports authors [28]. Most BRCA carriers in our study had an elevated level, which is in accordance with [29]. The majority of Algerian BRCA carriers and noncarriers in the present study were treated with mastectomy, due to lack of radiotherapy centers which constitute a great problem for breast cancer patients.

## 5. Conclusion

Our findings suggest that frequency of BRCA1/2 mutations may be more present in Algeria.* BRCA1/2* mutations are responsible for a significant proportion of hereditary and sporadic breast cancer among Algerian young women. These results represent the first step in comprehension of BRCA mutational profile among Algerian early-onset breast cancer women; additional work remains to be done with a larger sample size to better understand the role of these two genes in Algerian breast cancer. The integration of genetic testing into oncology practice is becoming increasingly widespread in developed countries; we hope that it will be in our country as a routine test. It would be of interest to study the relation between mutation location and breast cancer risk. The implications of these findings in regard to genetic testing and counseling are substantial for patients and their relatives. Finally and most importantly, we suggest that Algerian population should develop breast cancer mutation database.

## Figures and Tables

**Table 1:** Clinical characteristics of study population: age at diagnosis, at menarche, at first pregnancy, and at first use of oral contraceptive.

Mean age (years)	BRCA mutation carriers			Noncarriers
	BRCA1	BRCA	BRCA2	
At diagnosis	37.75	34.63	31.50	37.06

At menarche	15.00 ≤12: 00% 13-14: 25% 15-16: 75%	13.63 ≤12: 14.2% 13-14: 21.3% 15-16: 21.4%	12.25 ≤12: 50% 13-14: 50% 15-16: 00%	14.13 ≤12: 15.7% 13-14: 40.6% 15-16: 40.7% >16: 03.1%

At first pregnancy	22.25	24.00	26.33	26.50

At first use of oral contraceptive	26.00	26.29	26.66	26.15

**Table 2:** Genetics and histopathological characterizations of the Algerian BRCA mutation carriers.

Patients	Genes	Age at diagnosis (years)	Nucleotide change (HGVS)	Amino acid change	Variant type	Location Exon/Intron	Hormonal receptors		HER2 status	Tumor histotype/grade	Affected family members	
							RE	RP			Breast/ovarian cancers (age at diagnosis: years)	Other cancer (age at diagnosis: years)
801	BRCA1	40	c.1817del	(p.Pro606Leufs*∗*6)	FS	11	−	−	**−**	IDC/III	Sister, Br breast (38) Cousin P, breast (45)	No

860	BRCA1	39	Del exon 2		LGR	2	−	−	**−**	IDC/II	No	No

879	BRCA1	38	c.4065_4068del	(p.Asn1355Lysfs*∗*10)	FS	11	−	−	**−**	IDC/II	No	Uncle P, CUPS (70)

900	BRCA1	34	c.5332+1G>A		Splice donor variant	21	−	−	**−**	IDC/III	No	Grandmother M, cervix uteri (80) Aunt P, stomach (85) 2 cousins P, blood (40, 50) 2 uncles P, gastric (60, 65)

903	BRCA2	28	c.7654dupA	(p.Ile2552Asnfs*∗*2)	FS	16	+	+	**+**	IDC + ILC/II	Grandmother P, breast (65)	Father, brain (66)

911	BRCA2	38	c.1528G>T	(p.Glu510*∗*)	NS	10	+	+	**−**	IDC/II	No	Uncle M, colon (50)

988	BRCA2	30	Del exons 19-20		LGR	19/20	+	+	**−**	IDC/I	No	No

1000	BRCA2	30	c.6450del	(p.Val2151Phefs*∗*17)	FS	11	+	+	**−**	IDC + ILC/III	No	Cousin P, bone (23). Grandmother, blood (76)

FS: frameshift; NS: nonsense; LGR: large genomic rearrangement; IDC: invasive ductal carcinoma; ILC: invasive lobular carcinoma; M: maternal; P: paternal; CUPS: cancer of unknown primary site.

**Table 3:** Nonpathogenic variants found among younger Algerian women.

Patients	Genes	Age at diagnosis	Sequence variant	Consequence	Interpretation	Affected member family	
						Breast or ovarian cancers (age at diagnosis: years)	Other cancer (age at diagnosis: years)
760	BRCA1	39	c.5117G>C	(p.Gly1706Ala)	FP	No	Uncle M, liver 2 aunts P, blood (75, 80)
769	BRCA2	40	c.7462A>G	(p.Arg2488Gly)	VUCS	Mother, breast (62)	No
772	BRCA2 BRCA1	39	c.1504A>C	(p.Lys502Gln)	VUCS	Sister, breast (23)	No
			c.5117G>C	(p.Gly1706Ala)	FP		
789	BRCA2	40	c.5939C>T	(p.Thr1980Ile)	VUCS	No	No
798	BRCA2	39	c.1627C>A	(p.His543Asn)	VUCS	No	Father, lung (76)

FP: favor polymorphism, M: maternal, P: paternal, VUCS: variant of uncertain clinical significance.
